# Seborrheic keratoses: a distinctive diagnoses of pigmented vulvar lesions: a case report

**DOI:** 10.1186/1757-1626-3-56

**Published:** 2010-02-10

**Authors:** Esra Aktepe Keskin, Canan Gorpelioglu, Evren Sarifakioglu, Hasan Kafali

**Affiliations:** 1 Department of Gynecology and Obstetrics, Fatih University Faculty of Medicine, Ankara, Turkey; 2 Department of Dermatology, Fatih University Faculty of Medicine, Ankara, Turkey

## Abstract

Seborrheic keratoses, a benign growth lesion, is a very common cutaneous lesion encountered in white races in the fourth and fifth decade. The occurrence of this lesion on the vulva is rare, as an isolated lesion or in association with lesions elsewhere. A 34-year-old woman reported with a hyperpigmented palpable lesion, approximately 5-10 mm in diameter, was found on the patient's left labium majus. The clinical differential diagnosis of the pigmented lesions of the vulva is difficult often need a biopsy.

## Introduction

Seborrheic keratoses (SK) are common benign pigmented lesions on the light-exposed skin in white races, generally believed to be readily identifiable clinically, but in some cases, the differential diagnosis between pigmented SK, malignant melanoma and genital wart is difficult, in particular in the vulva [1,2].

## Case presentation

**Figure 1:**
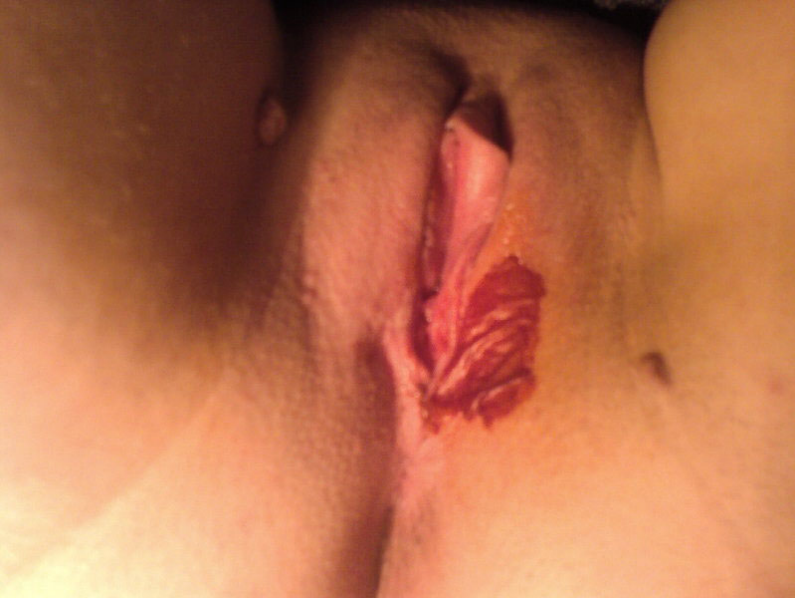
**Hiperpigmented lesion on the patient's left labium majus**.

**Figure 2:**
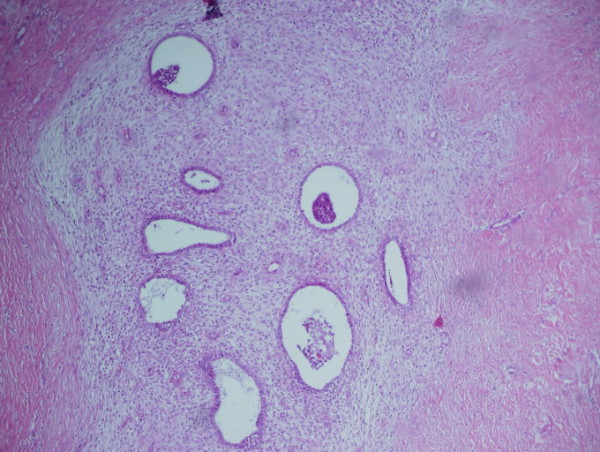
**Hystopathologic specimen clearly shows broad columns of highly pigmented basaloid cells intermingled with horn cysts (HE × 20)**.

A 34-year-old woman referred to the gynecology clinic with a complain about vulvar irritation due to a pigmented vulvar lesion. The patient reported that it had been present for approximately 1 year. On clinical examination, a hyperpigmented irregular palpable lesion, approximately 5-10 mm in diameter, was found on the patient's left labium majus (Figure 1). The initial clinical diagnosis was that of a genital wart but the lesion was suspicious therefore a punch biopsy was performed. Histopathological examination of the lesion, showed benign squamoid proliferation with acanthosis, hyperkeratosis and horny epidermal cells with pseudocystic inclusions of keratinized material (Figure 2). The patient was diagnosed with seborrheic keratoses of the vulva and cryotherapy was performed. The lesion disappeared from sight on control examination on 4 week later.

## Discussion

The vulva is a histological combination of skin and mucous membranes components. Pigmented vulvar lesions, including diffuse hyperpigmentation, are present in 10 to 12% of white women [3]. Some of vulvar pigmented lesions are tumoral, melanocytic or non-melanocytic, others non-tumoral, related to inflammatory, immunological, hormonal, or paraneoplasic mechanisms. The differential diagnosis of pigmented vulvar lesions includes vulvar intraepithelial neoplasia, epidermal nevus, wart, squamous cell carcinoma, condyloma accuminata, malignant melanoma, and verrucous carcinoma. The classical clinical features of SK (distinct keratotic and folicular plugging, stuck-on appearance, etc.) disappear because of the friction and maceration in the intertriginous region [2].

Dermoscopy is an useful method for the differential diagnosis of pigmented lesions even in the vulva. However, it is technically difficult and uncomfortable to use dermoscopy specially in this particular site. With the development of digital image systems with smaller and easy to handle probes this difficulty is overcomed [2]. Dermoscopy could not be performed in our patient owing to the fact of these difficulties in this particular site and that the patient was first refered to gynecology department and the biopsy was already taken.

These vulvar SKs are composed of cytologically bland, basaloid cells that do not exhibit koilocytotic atypia but do exhibit hyperkeratosis, papillomatosis, acanthosis, variable melanin pigmentation, and horn pseudocyst formation [4]. There is no evidence koilocytotic changes in our patient. Seborrheic keratoses is a tumoral, non- melanocytic and benign lesion which occurs on the light-exposed skin specially in elderly individuals. The occurrence of this lesion on the vulva is rare as an isolated lesion or in association with lesions elsewhere. Its percentage is unclear [5].

## Conclusion

Assessment of pigmented vulvar lesions necessitate sophisticated examination. The vulvar region must be carefully inspected by dermatologists and gynecologists. Moreover genital region must be included. Vulvar biopsies are suggested in suspicious lesions due to probability of malign melanoma.

To conclude, we think that in the differential diagnosis of vulvar pigmented lesions it should be keep in mind rarely this pigmented vulvar seborrheic keratoses.

## Consent

Written informed consent was obtained from the patient for publication of this case report and accompanying images. A copy of the written consent is available for rewiev from the journal's Editor-in-Chief.

## Competing interests

The authors declare that they have no competing interests.

## Authors' contributions

EAK performed first medical exam reviewed the literature and wrote the manuscript. CG and ES consultated the patient and took the biopsy. HK revisied the manuscript for important content. All authors read and approved the final manuscript.
